# Supplementary figures and images for: Variation in partner benefits in a shrimp—sea anemone symbiosis
Source: PeerJ. 2015 Nov 19;3:e1409. doi: 10.7717/peerj.1409 (PMC4655092; doi:10.7717/peerj.1409)

Normal Q-Q Plot

Sample Quantiles

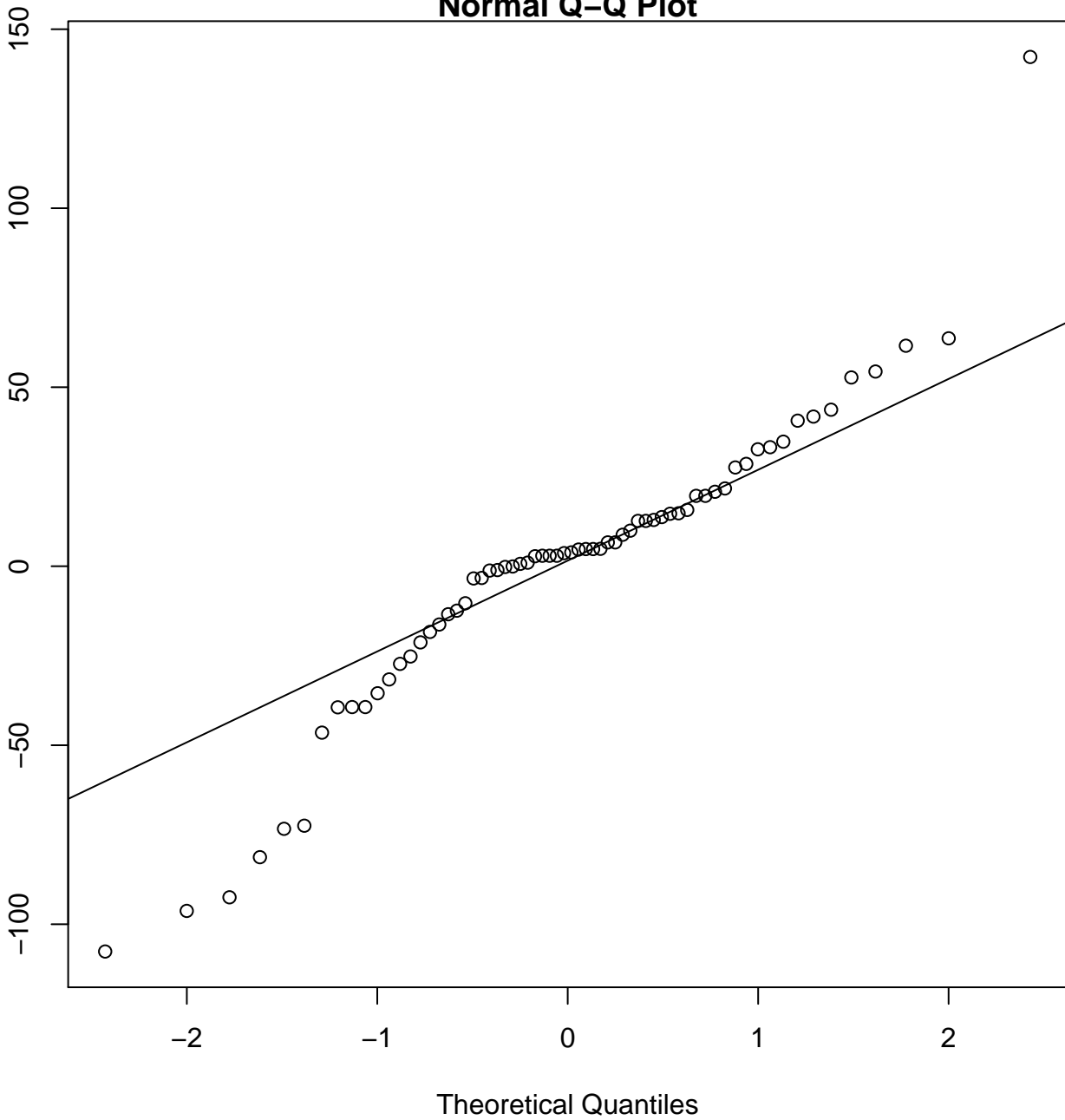

Theoretical Quantiles

Supplement: Figure S1 — Sample quantiles of residuals from the linear model of the number of eggs carried by an individual shrimp as a function its carapace length (F (1,64) = 81.71, p < 0.0001, R2 = 0.5608, Fig. 2), plotted against theoretical quantiles under the assumption of normality. [file peerj-03-1409-s003.pdf]
